# Exercise stress CMR reveals reduced aortic distensibility and impaired right-ventricular adaptation to exercise in patients with repaired tetralogy of Fallot

**DOI:** 10.1371/journal.pone.0208749

**Published:** 2018-12-31

**Authors:** Paul Habert, Zakarya Bentatou, Philippe Aldebert, Mathieu Finas, Axel Bartoli, Laurence Bal, Alain Lalande, Stanislas Rapacchi, Maxime Guye, Frank Kober, Monique Bernard, Alexis Jacquier

**Affiliations:** 1 Aix-Marseille Univ, CNRS, CRMBM, Marseille, France; 2 Department of Radiology and Cardiovascular Imaging, La Timone Hospital, Marseille, France; 3 Department of Cardiology and Department of Infectious Diseases, La Timone Hospital, Marseille, France; 4 Department of Vascular Surgery and Vascular Medicine, La Timone Hospital, Marseille, France; 5 LE2I, UMR 6306 CNRS, University of Burgundy, Dijon, France; 6 MRI Department, University Hospital of Dijon, Dijon, France; Faculty of Medical Science - State University of Campinas, BRAZIL

## Abstract

**Background:**

The aim of our study was to evaluate the feasibility of exercise cardiac magnetic resonance (CMR) in patients with repaired tetralogy of Fallot (RTOF) and to assess right and left ventricular adaptation and aortic wall response to exercise in comparison with volunteers.

**Methods:**

11 RTOF and 11 volunteers underwent prospective CMR at rest and during exercise. A supine bicycle ergometer was employed to reach twice the resting heart rate during continuous exercise, blood pressure and heart rate were recorded. Bi-ventricular parameters and aortic stiffness were assessed using accelerated cine sequences and flow-encoding CMR. A t-test was used to compare values between groups. A Mann Whitney test was used to compare values within groups.

**Results:**

In RTOF both ventricles showed an impaired contractile reserve (RVEF rest 36.2±8.3%, +1.3±3.9% increase after exercise; LVEF rest 53.8±6.1%, +5.7±6.4% increase after exercise) compared to volunteers (RVEF rest 50.5±5.0%, +10.4±7.1% increase after exercise, p = 0.039; LVEF rest 61.9±3.1%, +12.2±4.7% increase after exercise, p = 0.014).

RTOF showed a reduced distensibility of the ascending aorta during exercise compared to volunteers (RTOF: 3.4±1.9 10-3.mmHg^-1^ vs volunteers: 5.1±1.4 10-3.mmHg^-^^1^; p = 0.027). Ascending aorta distensibility was correlated to cardiac work in the volunteers but not in RTOF.

**Conclusion:**

RTOF showed an impaired contractile reserve for both ventricles. The exercise unmasked a reduced distensibility of the ascending aorta in RTOF, which may be an early sign of increased aortic rigidity.

## Introduction

Cardiac magnetic resonance (CMR) is particularly valuable in the assessment of diseases that affect the right heart. CMR is a validated tool that is widely accepted for monitoring biventricular volumes and function in patients with repaired tetralogy of Fallot (RTOF) [1, 2]. CMR permits highly reproducible, accurate measurements of RV volumes and function even during exercise [3]. To date rest right ventricular (RV) end diastolic volume (EDV) assessed using CMR is a major parameter used to assess the right timing for pulmonary valve replacement after surgery in childhood [4]. Concomitant stress may unmask latent pathology and provide incremental information to assess prognosis and guide therapy [5]. For instance, low-dose dobutamine stress has been shown to unmask RV dysfunction in RTOF [6]. Less invasive and more reproducible than pharmacological stress, exercise-induced changes may be helpful to identify left and right ventricular heart disease that is not apparent at rest [7]. It may also be useful in the assessment of RV contractile reserve. Apart from preliminary work using CMR, the assessment of biventricular function at rest or during exercise was restricted to the use of radionuclide angiography [8] or echocardiography [9]. Dedicated CMR protocols have been optimized to study cardiac function during supine submaximal bicycle exercise [10]. Exercise stress CMR can be performed either using a non-magnetic ergometer directly on the CMR table or on a treadmill [11, 12]; this method has proven promising in the evaluation of myocardial ischemia, since its equivalence to myocardial scintigraphy has been demonstrated [13].

Tetralogy of Fallot is a cyanotic congenital heart disease [14] and requires corrective surgery early in life to improve long-term survival [4]. CMR is the method of choice for assessing pre-operative parameters in RTOF [15, 16]. Exercise tests on patients with RTOF are possible [17], but very few studies on RTOF patients’ response to exercise have measured bi-ventricular parameters [18, 19], and, to the best of our knowledge, none has explored aortic compliance (AC).

CMR has been described as an effective tool to assess aortic distensibility (AD) at rest, showing a strong correlation with pulse wave velocity, in healthy, aging populations and patients with Marfan syndrome [20–22]. Lalande et al. demonstrated that the compliance impairment of the ascending aorta could serve as an early marker of changes in the aortic wall, with a discriminative value in asymptomatic patients, independently of the presence of aortic dilatation [23, 24].

The aims of our study were 1) to assess the feasibility of exercise stress cardiac magnetic resonance in RTOF and volunteers 2) to assess biventricular parameters and aortic rigidity in RTOF at rest and during exercise stress CMR in comparison to volunteers.

## Materials and methods

### Subjects

Informed written consent was obtained from all patients and volunteers in accordance with the Declaration of Helsinki after approval of the protocol by the University Hospital’s ethics committee. Eleven patients with RTOF and eleven volunteers were studied (Table 1). Inclusion criteria were the need to perform a CMR exam in the follow-up of the pathology, absence of medical contraindications to physical exercise and the ability to hold their breath for approximately twenty seconds. Exclusion criteria were presence of an implanted cardiac device, history of heart rhythm disorder or heart failure or contraindications for CMR. Eleven volunteers were included as a control group with the following inclusion criteria: normal electrocardiographic findings at rest, no history of cardiovascular disease, the ability to undergo stress exercise and no cardiac symptoms. Exclusion criteria were presence of cardiac malformations and contraindications for CMR (Table 1).

**Table 1:** Patients characteristics.

	RTOF	Volonteers	p
Sex (F/M)	5/6	4/7	
Age (y)	37.3±13.8	29.1±4.7	ns
Weight (kg)	65.8±11.6	64.9±9.8	ns
Height (m)	1.7±0.1	1.7±0.1	ns
BMI (kg/cm^2^)	22.7±2.5	20.7±2.7	ns
Body area (m^2^)	1.6±0.2	1.6±0.2	ns
Age of surgery (y)	9.9±8.9		
Patch necessary	7/11		
Palliative shunt needed	5/11		

BMI = body mass index. RTOF = repaired tetralogy of Fallot

### CMR protocol

All subjects underwent CMR on a 1.5T magnet (Siemens, Avanto, Erlangen, Germany) using a 16 channel anterior surface coil array and a 9-element posterior spine-coil array. The supine bicycle exercise stress and CMR imaging protocol were described and validated in detail previously [25]. All images at rest and during stress were acquired during short breath-holds. For ascending aortic analysis, steady state free precession cine images were acquired at the level with the pulmonary trunk. The imaging plane was positioned perpendicular to the ascending aorta (AAo) and the proximal descending aorta (DAo) so that both could be measured on the same image. The difference in angulation between AAO and DAo never exceeded 15°. The following parameters were used: TR = 21.7ms, TE = 1.36ms, α = 65°, FOV = 280×340, slice thickness = 7 mm, pixel size = 1.26×1.26mm, GRAPPA 2, 30 to 40 phases were reconstructed. For the ventricular parameters, a short-axis balanced steady state free precession cine sequence with TR/TE of 2.3/1.0ms and a GRAPPA acceleration factor of 3 was used for imaging cardiac function. Additionally, 2 and 4 chamber cine sequence were acquired at rest.

Velocity encoding gradient pulse sequences were acquired to assess the regurgitant fraction of the pulmonary valve and were positioned perpendicular to the pulmonary artery. The following parameter were used: through plane direction, maximum encoding velocity 200 cm/s, TR = 28.35ms, TE = 2ms, α = 30°, slice thickness = 6 mm, FOV = 192x280, 60 reconstructed phases. Data were reconstructed in magnitude and phase. If flow artefacts were found on the SSFP images level with the ascending aorta, aortic compliance was measured by using magnitude images reconstruction acquired on the same plane as the cine images [23].

### Supine bicycle exercise in the CMR room

**Fig 1:**
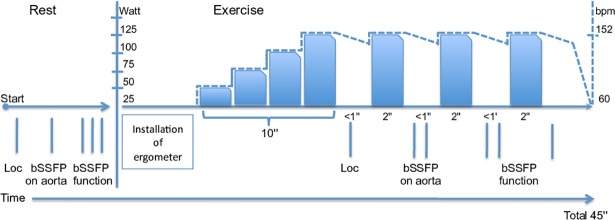
Diagram illustrating the progression of the cardiac imaging protocol. Goal was to obtain twice the resting heart rate (Note the right Y axis representing an example of one volunteer). Each vertical line represents an apnea.Cine for cardiac function lasting 3 min, carried out across three breath holds of about 12s. Each cine represents the average of 5 heartbeats, 4 slices are acquired in one breath hold. ‘Loc’: Localizer sequence. After exercise this was done only if necessary.

For CMR exercise, a non-magnetic ergometer (Lode B.V., Groningen, the Netherlands) was mounted on the patient table. The position of the ergometer foot pedals was adjusted to the height of the subject. The heart rate was measured by electrocardiography. The training mode of the ergometer allowed us to adjust the workload in Watts and was independent of pedal rotations per minute defining. Initial workload was 25 Watts. It was increased by 25W steps every 2 min, with a minimum duration of 10 min. The revolutions per minute ranged from 50 to 70 in order to ensure that work load was independent of revolution per minute. Once the targeted heart rate was reached (double of the heart rate at rest), the subject was instructed to stop cycling and the table was moved to the center of the magnet for imaging. Considering that the heart rate and blood pressure diminished quickly, only two cine datasets were acquired immediately after the cycling stopped. The exercise heart rate was recorded at the time of imaging during each image acquisition. The exercise heart rate was calculated as the mean heart rate value measured during the first cine and the second cine data set acquired during the same exercise stop. The delay between the end of exercise and image acquisition was 20±7s. Before and after each exercise stop, the arterial diastolic and systolic blood pressures were measured using a brachial automatic sphygmomanometer (Maglife, Schiller Medical). The exercise blood pressure reported in the result section was the mean systolic and the mean diastolic pressure calculated during an exercise stop. The subject was then again moved out of the magnet to resume cycling, starting at the last exercise level for at least two minutes. The stress process was repeated until completion of stress CMR (Fig 1). Cardiac work was evaluated using the rate pressure product (RPP = systolic blood pressure x heart rate) [26, 27].

### Data post processing

Argus software (Siemens Healthineers, Erlangen, Germany) was used to measure all ventricular parameters, i.e. ejection fraction, end diastolic/systolic volume, stroke volume (SV), cardiac output (CO) at rest and during stress. The segmentation was performed using a semi-automatic method initiated by visually selecting the end diastolic and end systolic frames, and manually tracing the endocardial borders. All data were analyzed by consensus between a junior radiologist and a senior radiologist specialized in CMR with over 10 years of experience. Aortic compliance (AC) data were analyzed using the QIR scientific image analysis software (Université de Bourgogne, Dijon, France) [23]. The contours of the aorta were automatically traced for all phases of the cardiac cycle. For this processing [28] we used the balanced steady state free precession cine images because these images provided a high degree of contrast between the aortic wall and lumen with manageable artifacts due to flow. For two RTOF, these data were not available due to flow artifact within the aortic lumen and were successfully replaced by flash cine images. User interaction was limited to the indication of only one point near the center of the aorta on the first time frame. The AC is the absolute change in aortic area (Δarea) for a given pressure step (AC = Δarea/ΔP). Aortic distensibility (AD) was defined as the compliance divided by the minimum cross-sectional area. AC and AD rely on cross-sectional areas.

### Statistical analysis

Continuous data with a normal distribution are expressed as mean±SD and non-normal distributed data as median (range). Categorical data are expressed as frequency or percentage. All data were analyzed with Prism 5 (GraphPad Software, Inc, San Diego, CA, USA). A non-parametric Kruskal-Wallis test followed by a post-hoc Bonferroni test was performed to compare ventricular parameters, AC and AD between exercise and rest, for RTOF versus volunteers. A Mann Whitney test was used to compare all values at rest and exercise within groups. Correlation between AC, AD and biventricular parameters were checked with a Spearman test. A p-value below 0.05 was considered to indicate statistical significance.

## Results

### Study population and exercise test

The characteristics of the cohorts are shown in Table 1. There was no significant difference in age, weight, height, BMI and body surface between groups. One patient was excluded before CMR because of arrhythmia. All RTOF and volunteers completed the protocol. All CMR images were of good quality in both the volunteers and the patients. All subjects included in the study complete the CMR protocol in 45±5 min, all patients and volunteers were able to exercise and double their heart rate during exercise. Also, the exercise doubled the rest RPP in both RTOF (97.8 ± 39.4%) and volunteers (108.3 ± 55.8%) (Table 2). There was no statistical difference between the two groups’ rest and exercise parameters (Resting RPP and heart rate were respectively: 7 731±1 568 mmHg.min^-1^ versus 8 321±2 125 mmHg.min^-1^, p = 0.49; 69.6±10.3 bpm versus 68.4±13.6 bpm, p = 0.93; on exertion 15 412±4 844 versus 16 800±3 742 mmHg.min^-1^, p = 0.48 and 108.7±18.8 bpm versus 110.8±27.5 bpm, p = 0.82) (Table 2). In the RTOF patients the mean regurgitant fraction of the pulmonary valve was measured at 25.9±23.9%.

**Table 2:** Hemodynamic parameters.

	RTOF		Volunteers		
	Rest	exercise	Rest		exercise
RPP (mmHg.min^-1^)	7731±1568	15412±4844*	8321±2125		16800±3742*
Heart rate (bpm)	69.6±10.3	108.7±18.8*	68.4±13.6		110.8±27.5*
SBP (mmHg)	114.3±16.2	143.1±28.4*	123.2±13.5		154.9±11.6*
DBP (mmHg)	74.2±9.3	84.1±22.3	70.7±7.0		74.2±7.6
PP (mmHg)	40.1±7.7	59.2±6.3*	53.9±6.6§		80.3±4.7*§
MBP (mmHg)	87.2±10.1	103.2±23.5*	87.5±8.8		100.0±8.8*

RTOF = repaired tetralogy of Fallot. RPP = rate pressure product. SBP = systolic blood pressure. DBP = diastolic blood pressure. PP = pressure pulsed. MBP = mean blood pressure.

*, significant *P* value for rest *vs* exercise.

§, significant P value for RTOF vs volunteers.

### Ventricular parameters at rest and during exercise

**Fig 2:**
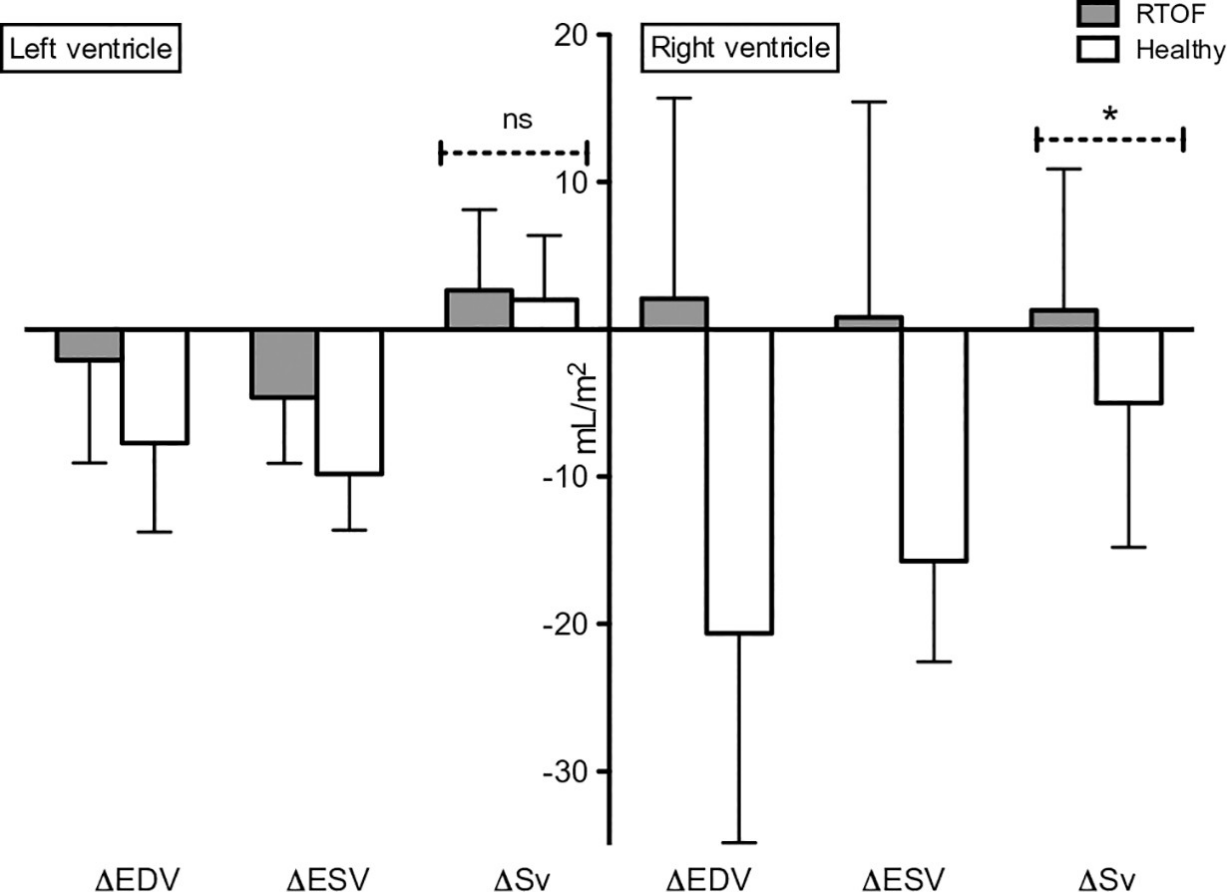
Change in ventricular parameters between stress and rest. Δ = exercise volume value minus rest volume value. EDV = end diastolic volume. ESV = end systolic volume. Sv = stroke volume. *: p = 0.028. ns = non-significant p-value. All DATA are indexed to BSA. There was a significant difference in right ventricular stroke volume, because patients do not adapt their ventricular muscle to exercise. Even if there was no other statistical difference we observed a tendency to lower biventricular contractility in patients.

**Fig 3:**
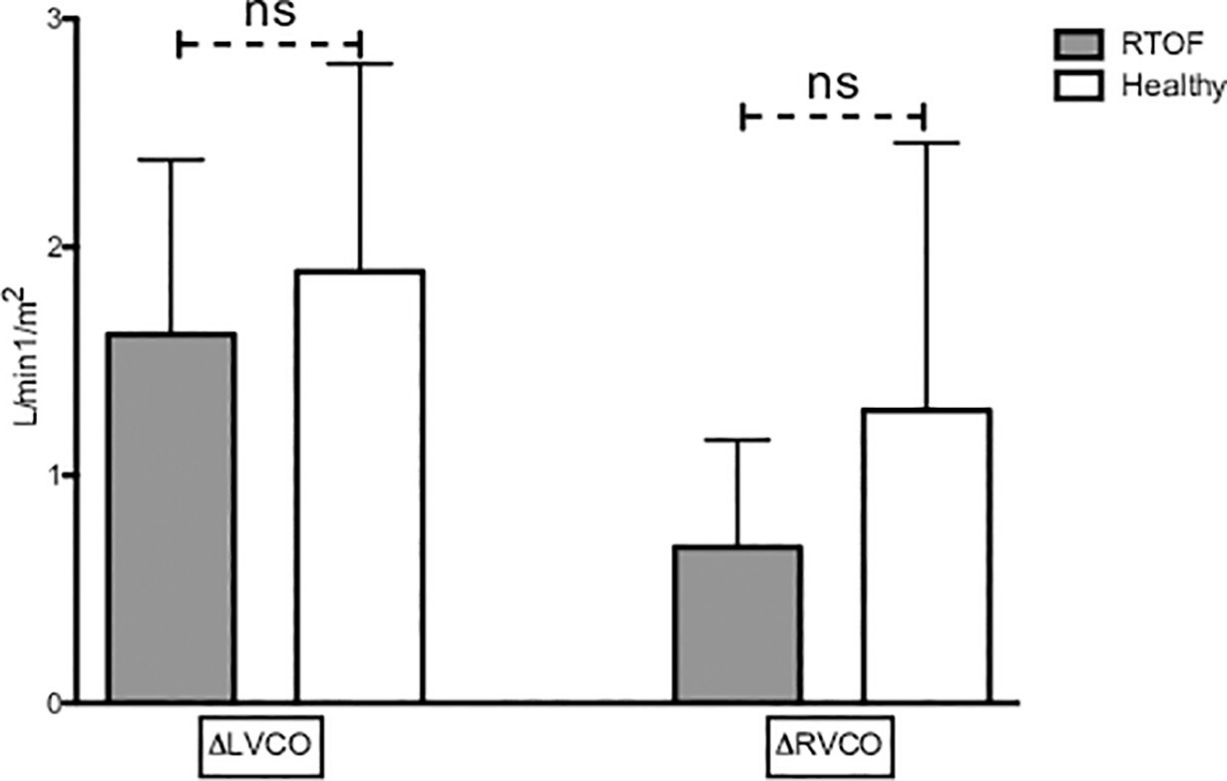
Change in cardiac output during exercise and at rest. Δ = exercise percentage value minus rest percentage value. LVCO = left ventricular cardiac output. RVCO = right ventricular cardiac output. ns = nonsignificant p-value. This figure shows that there was no difference between the groups in terms of adaptability of the flow during exercise; the plots on the right side show that the RVCO tends to increase less in the RTOF.

**Fig 4:**
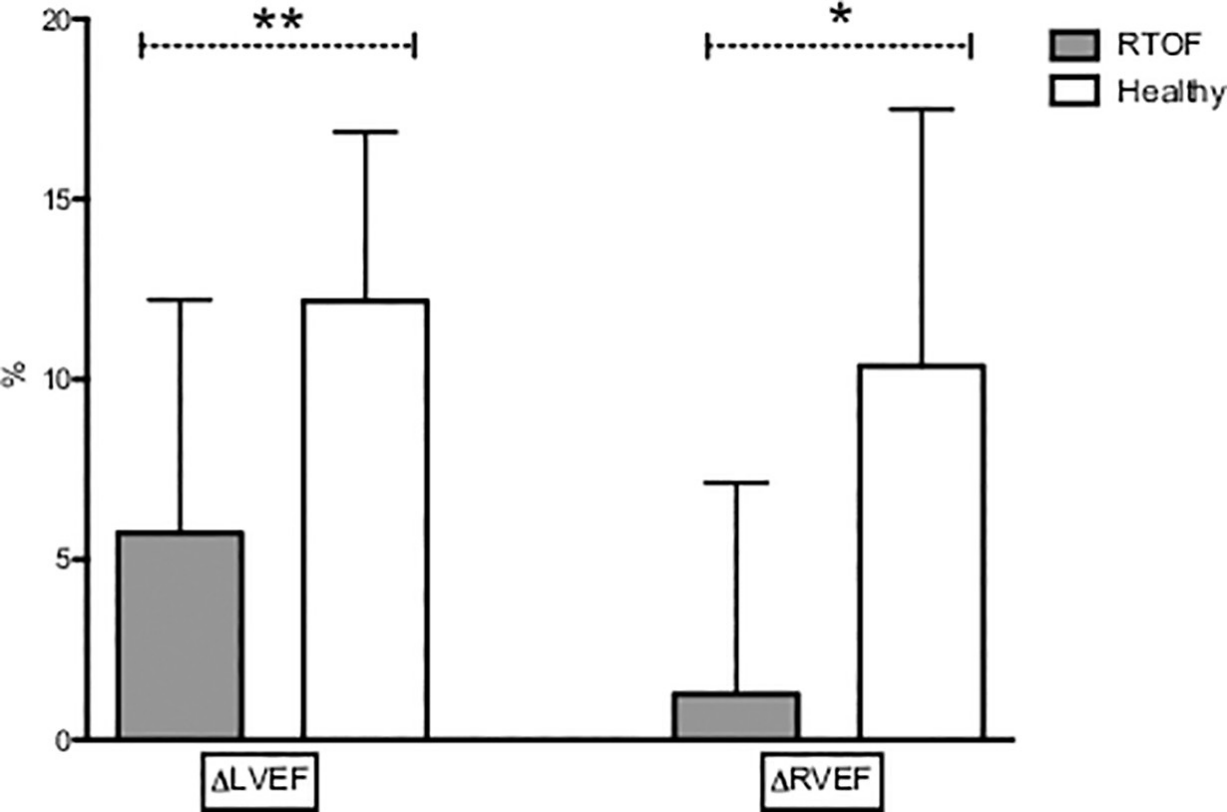
Change between exercise and rest for ejection fraction. Δ = exercise percentage value minus rest percentage value. LVEF = left ventricular ejection fraction. RVEF = right ventricular ejection fraction. *: p = 0.039. **: p = 0.014. This figure shows that patients do not adapt their ventricular function to exercise.

Rest LVEF was reduced for the RTOF (53.8±6.1%), but the increase in response to exercise was significant (59.6±10.4% p = 0.015). There were no significant changes in SV index between rest and exercise in either population (36.6±8.9 ml/m^2^ to 39.1±12.3 ml/m^2^, p = 0.14 for RTOF and 40.4±8.1 ml/m^2^ to 42.4±7.6 ml/m^2^ p = 0.24 in volunteers) (Fig 2). However, CO increased significantly during exercise (2.5±0.5 l/min/m^2^ to 4.1±1.1 l/min/m^2^, p = 0.001 for RTOF and 2.7±0.7 l/min/m^2^ to 4.6±1.1 l/min/m^2^, p = 0.001 in the volunteers) (Fig 3). The ability to adapt ventricular parameters to exercise represented by the change between the stress and resting values was not significantly different between the two groups for LVEF, SV and CO, reflecting LV adaptation to exercise in both groups. Resting LVEF was normal in volunteers and increase significantly in response to exercise (61.9±3% to 74.1±4.1%; p = 0.0038). The volunteers showed a greater capacity to adapt their LVEF than the RTOF, and this difference was significant (+12.2±4.7% versus +5.7±6.4% respectively, p = 0.014) (Fig 4).

Concerning the RV, the RTOF showed no increase in their ejection fraction between resting 36.2 ± 8.3% and exercise 37.2 ± 12.2% p = 0.48. The difference in RVEF between rest and exercise was higher for the volunteers compared with the RTOF (+ 10.4±7.1% vs + 1.3±3.9%; p = 0.039; respectively) (Figs 2 and 4). These parameters reflect an absence of contractile reserve of the RVEF in RTOF. As expected, the volunteers adapted their RV function and volume to stress (Table 3).

**Table 3:** Ventricular and aortic parameter.

	RTOF		Volonteers	
	Rest	exercise	Rest	exercise
LV				
EDVi (ml/m^2^)	68.3±17.6	65.9±22.4	64.8±11.0	57.1±9.5*
ESVi (ml/m^2^)	31.5±11.0	26.8±14.0*	24.6±4.0	14.7±3.2*
EF (%)	53.8±6.1	59.6±10.4*	61.9±3.0§	74.1±4.1*§
Svi (ml/m^2^)	36.6±8.9	39.1±12.3	40.4±8.0	42.4±7.6
COi (l/min/m^2^)	2.5±0.5	4.1±1.1*	2.7±0.7	4.6±1.0*
RV				
EDVi (ml/m^2^)	179.5±69.9	181.4±73.6	84.3±21.0§	63.6±24.3*§
ESVi (ml/m^2^)	117.7±53.3	118.6±61.9	40.8±6.7§	25.1±10.0*§
EF (%)	36.2±8.3	37.2±12.2	50.5±5.1§	60.8±4.1*§
Svi (ml/m^2^)	61.7±21.3	63.3±21.9	43.6±14.8	38.0±14.7§
COi (l/min/m^2^)	4.1±1.3	6.5±1.9*	2.9±1.1	4.2±1.8*
COi† (l/min/m^2^)	2.8±0.9	3.5±1.1*		
AC				
AAo (mm^2^.mmHg⁻^1^)	3.4±0.5	2.3±1.2*	2.8±0.8	2.1±0.7*
DAo (mm^2^.mmHg⁻^1^)	1.5±0.5	1.1±0.4*	1.4±0.3	0.9±0.3*
AD				
AAo (10⁻^3^.mmHg⁻^1^)	5.3±2.2	3.4±1.9*	6.5±1.5	5.1±1.4*§
DAo (10⁻^3^.mmHg⁻^1^)	7.1±2.1	5.3±1.9*	6.1±1.6	4.2±1.5*

RTOF = repaired tetralogy of Fallot. LV and RV = left and right ventricles. CO = cardiac output measure on cine function sequences. ESV = end systolic volume. EDV = end diastolic volume. Sv = Stroke volume. EF = ejection fraction. AC = aortic compliance. AD = aortic distensibility. i = index. AAo = ascending aorta. DAO = descending aorta. All measures are indexed.

†: CO measured with flow sequence, to avoid false value of CO induced by the pulmonary regurgitation fraction.

* Significance level of differences obtained between exercise and rest was p<0.05.

§ Significance level of differences obtained between RTOF and volunteers was p<0.05.

Cardiac work correlated significantly with right and left CO indexed for RTOF and volunteers. The correlation coefficient was lower for RTOF (right CO: R = 0.45 p = 0.044, left CO: R = 0.66, p < 0.001 for RTOF and right CO: R = 0.75 p < 0.001, left CO: R = 0.92, p < 0.001 for volunteers) reflecting a greater adaptation to exercise in volunteers compared with the RTOF group (Table 3).

### Aortic compliance and distensibility at rest and during exercise

**Fig 5:**
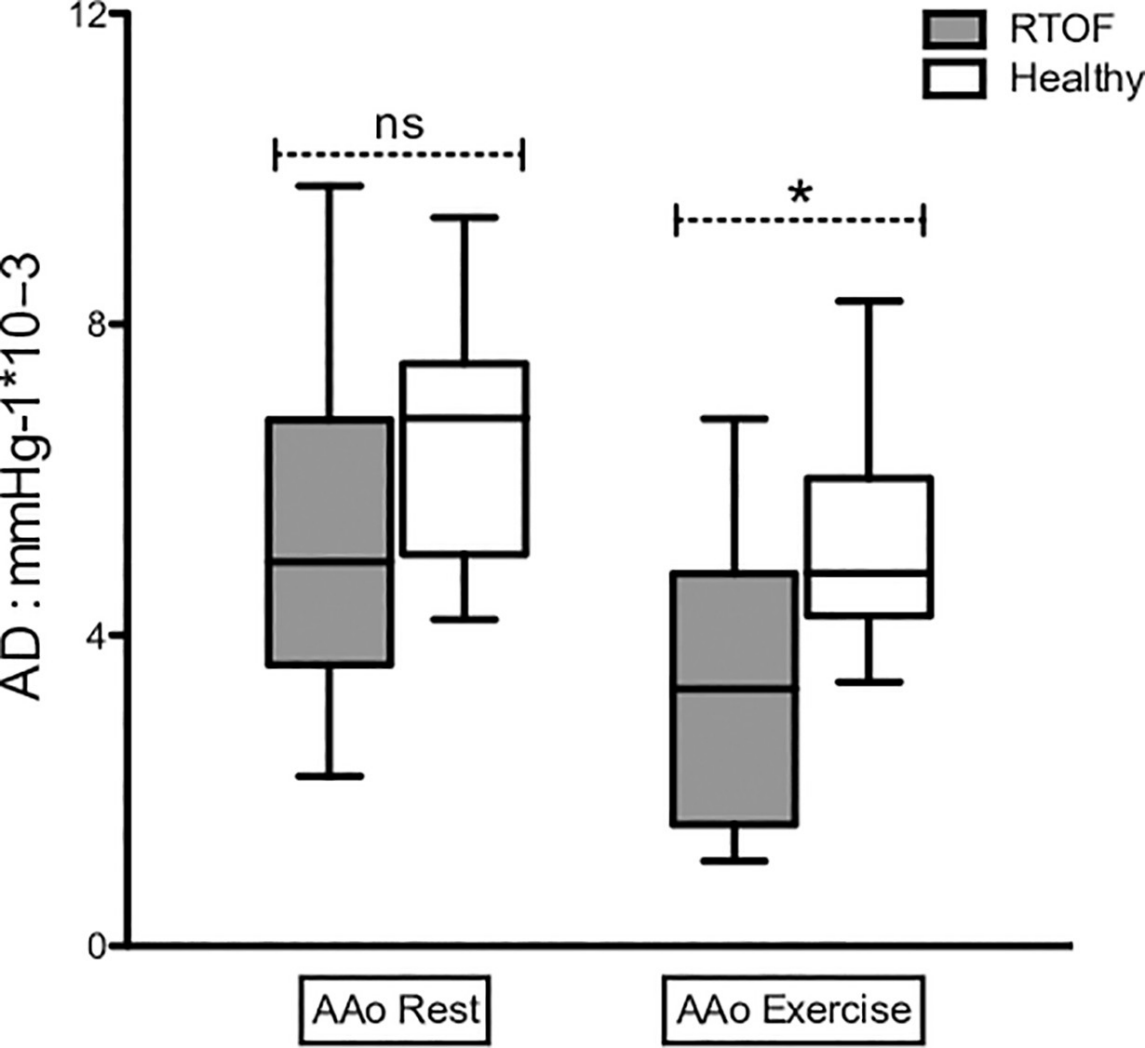
Comparison between patients and volunteers for aortic distensibility at rest and exercising. AD = aortic distensibility. AAo = ascending aorta. DAo = descending aorta. *: p = 0.027. ns = non-significant p-value. This result shows a statistical difference between the patients and volunteers on AAo when exercising which does not exist at rest. This suggests that exercise can reveal a decrease in aortic distensibility at an early stage.

**Fig 6:**
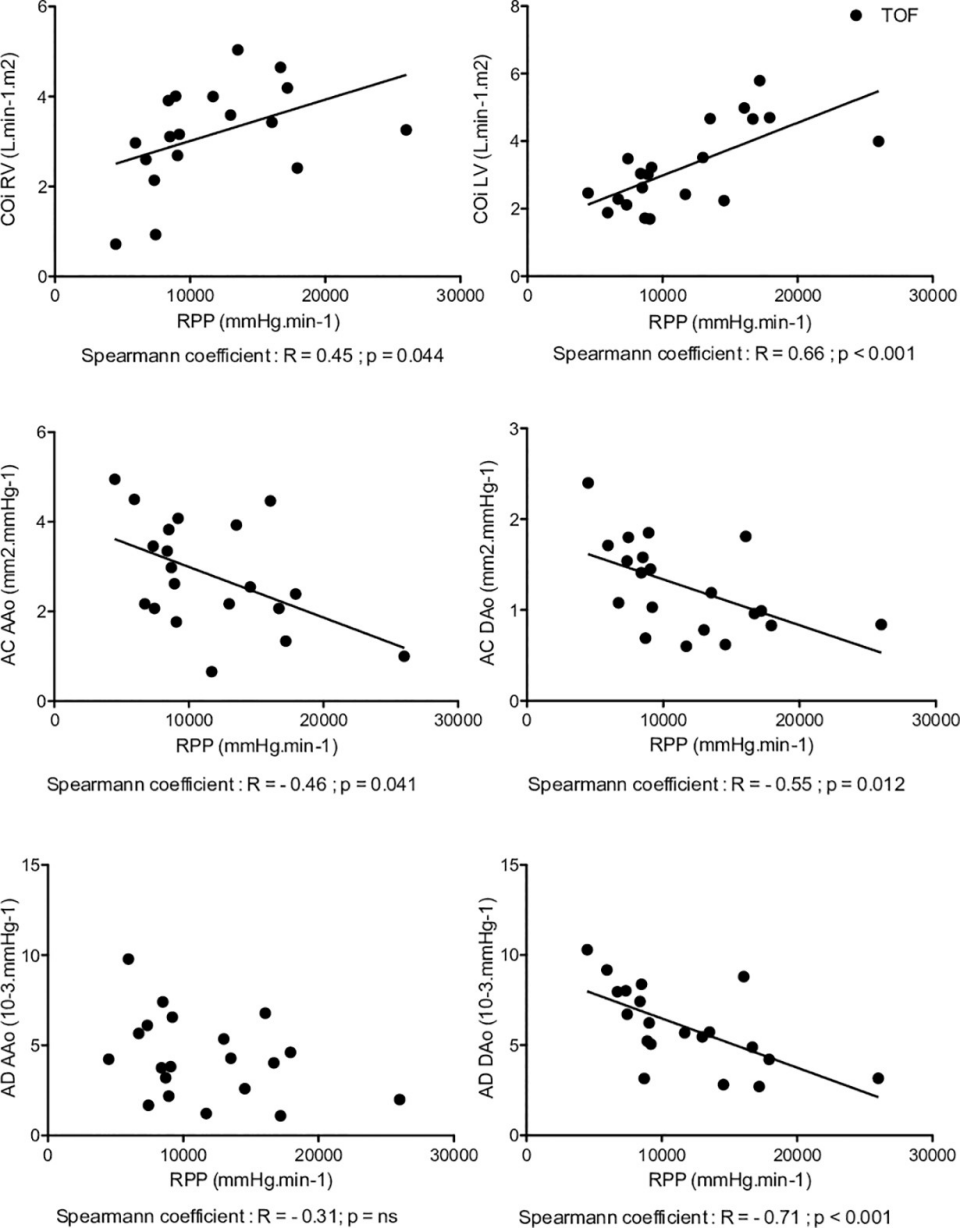
Correlation curve showing rate pressure pulse and parameters for repaired tetralogy of Fallot. LV and RV = left and right ventricles. CO = cardiac output. i = index. RPP = rate pressure pulse. AC = aortic compliance. AD = aortic distensibility. AAo = ascending aorta. DAO = descending aorta. All measurements are indexed. In RTOF at rest and on exertion a significant correlation was found between cardiac work and both biventricular cardiac output. This correlation is less stronger than these of volunteers. This clearly demonstrate a lack of adaptation of AD AAo with RPP.

**Fig 7:**
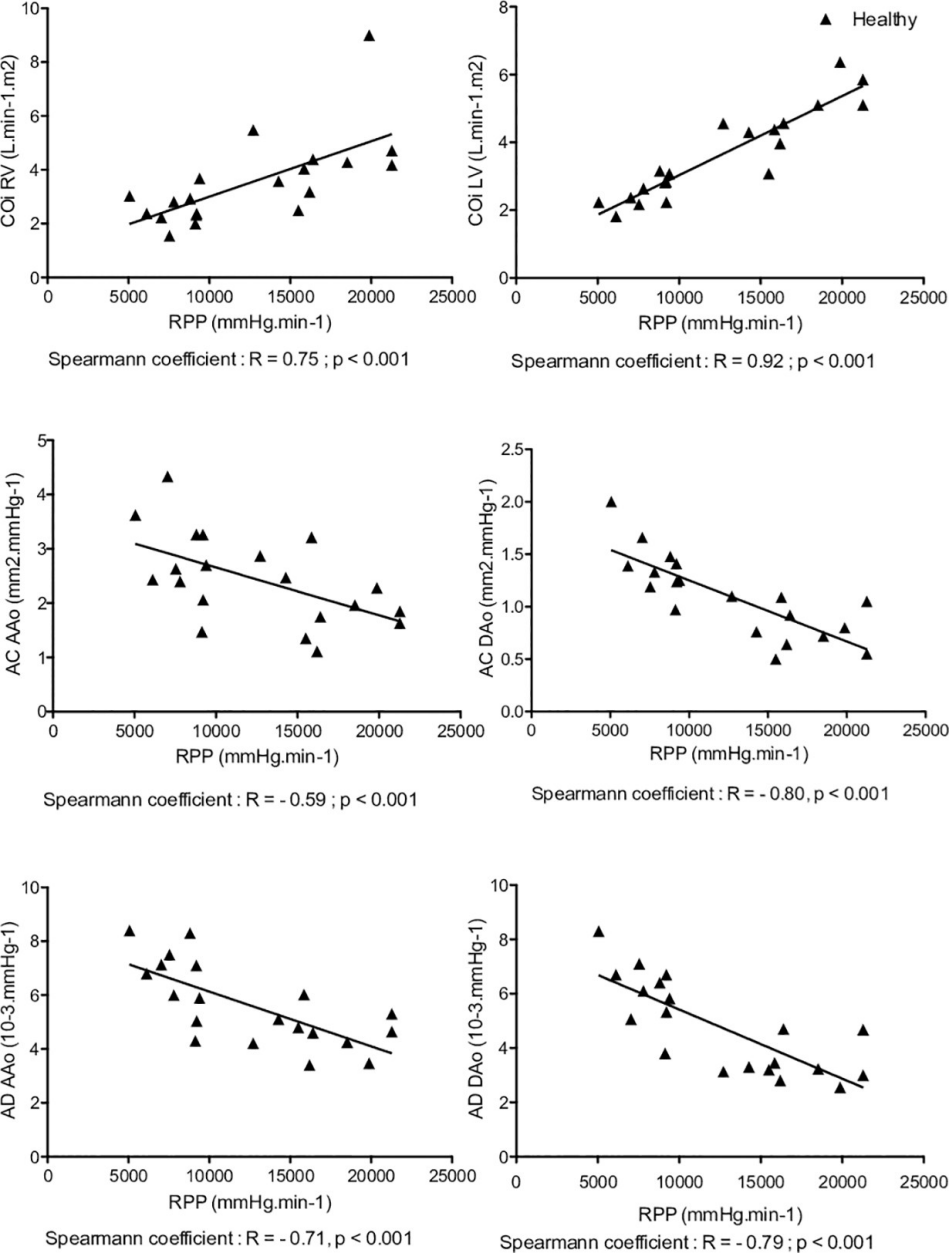
Curve showing rate pressure pulse correlation and parameters for volunteers. LV and RV = left and right ventricles. CO = cardiac output. i = index. RPP = rate pressure pulse. AC = aortic compliance. AD = aortic distensibility. AAo = ascending aorta. DAO = descending aorta. All measures are indexed. In volunteers at rest and on exertion a strong and significant correlation was found between cardiac work and both biventricular cardiac output; cardiac work and all the stiffness parameters.

AC and AD decreased significantly and similarly for the RTOF and volunteers during exercise, both on the AAo and on the DAo (Table 3). AD was significantly reduced in control on exertion (AAo: 6.5±1.5 10^-3^.mmHg^-1^ to 5.1±1.4 10^-3^.mmHg^-1^ p = 0.001 and DAo: 6.1±1.6 10^-3^.mmHg^-1^ to 4.2±1.5 10^-3^.mmHg^-1^). A statistical difference was found on the AD of AAo at exercise between groups (3.4±1.9 10^-3^.mmHg^-1^ for RTOF versus 5.1±1.4 10^-3^.mmHg^-1^ for volunteers, p = 0.027) (Fig 5). There was no significant difference in AD between exercise and rest in either group (ΔAD AAo -1.9±2.6 10^-3^.mmHg^-1^ for RTOF versus -1.4±0.9 10^-3^.mmHg^-1^ for the volunteers; p = 0.88; ΔAD DAo -2.1±2.3 10^-3^.mmHg^-1^ for RTOF versus -2.1±1.2 10^-3^.mmHg^-1^ for volunteers; p = 0.55). RPP was correlated with all parameters of aortic rigidity except for the AD of the AAo in RTOF (AD, R = -0.31; p = 0.18) (Fig 6). Whereas a significant correlation for the AD of the AAo was observed in the control group (R = -0.71, p <0.001). We noted a much stronger correlation between the RPP and all the parameters of aortic stiffness in the control group (Fig 7).

## Discussion

This study showed that an exercise CMR using an ergometer is feasible within a reasonable delay in patients with RTOF operated in childhood. The exercise protocol was sufficient to double the resting RPP. The exercise stress CMR revealed a lack of contractile reserve of the RV in the RTOF. The study also showed a decrease in AC and AD during exercise in both groups but with no correlation between AD and RPP in RTOF. The exercise may therefore be a marker indicating a structural modification of the aortic wall. CMR is the reference method to quantify RV volumes and allow to assess cardiovascular adaptation to exercise in RTOF. More study will be needed to assess the impact of such parameters on patient selection for pulmonary valve surgery.

### Exercise stress CMR

Exercise CMR can also be performed using a treadmill in the examination room next to the scanner. In this setting the patient exercised first and is moved to the examination table immediately after completion of the exercise phase [29]. However, with this protocol, a lot of time was lost installing the patient on the CMR table and even more so in debilitated patients with right ventricular dysfunction. In our study we chose a protocol using an ergometer directly on the CMR table to avoid the previous limitations [30, 31]. In our setting the patient or volunteers had to cycle in a supine position. Exercise in the supine position did not develop the same power as on a treadmill. However, we showed in our study that both groups succeeded in doubling their RPP. Le et al. performed a free breathing exercise test without stopping pedaling, and showed that it was possible to calculate ventricular parameters [32]. The bore size of their magnet was 10 centimeters wider than ours, allowing legs motion within the magnet. Exercise stress CMR requires caution as a stress test; it entails the same risks as an electrocardiographic exercise test or single photon emission computed tomography [29, 33].

The RPP is a documented and reliable tool for evaluating cardiac stress during physical activity [34]. Based on established standards, rest activity was around 7 524 mmHg.min^-1^, sub-maximal exercise at 21 218 mmHg.min^-1^ and maximum exercise at 32 798 mmHg.min^-1^ [35]. The resting RPP in patients and in volunteers was at 7 731 mmHg.min^-1^ and 8 321 mmHg.min^-1^ respectively within the standards. However, the RPP values during the exercise were at 15 412 mmHg.min^-1^ and 16 800 mmHg.min^-1^ in patients and controls respectively, this was in line with our objective to explore adaptation to exercise and not to test the patient’s peak exercise capacity.

It accepted that CMR can be more reproducible in the analysis of the left ventricle on exertion than echocardiography. Also, an exercise stress CMR took more or less the same time to perform as exercise echocardiography [36]. Our study corroborated exercise CMR’s potential capacity to assess both ventricles and the aortic arch within a similar time frame. CMR also provided good quality images in all volunteers as well as in patients.

### Ventricular parameters

Baseline RVEDVi and RVESVi in the RTOF group were severely increased indicating that patients showed severe RV remodeling. In that group during exercise no increase in RVEDVi nor RVESVi was measured indicating a lack of contractile reserve in the right ventricle. The increase in cardiac output during exercise was only due to the heart rate increase. In the RTOF subjects LV parameters were preserved at rest but during exercise the LV showed a lower decrease of LVEDVi compared to the volunteers suggesting that LV contractile reserve is also impacted by the severe RV impairment. Adaptation of the RV to exercise in RTOF had not yet been extensively explored. However, a loss of adaptation of the RVEF is well documented [37] and has identified the pulmonary infundibulum, in particular in the anterior and lateral portion, to be the anatomical region which contracts the least. The study of Wald et al. Using CMR reported contrast uptake in these areas using CMR, suggesting a fibrous evolution of the myocardium [38].

In volunteers, exercise modification was in accordance with the literature reporting cardiac elongation force [39–41], namely a decrease in end diastolic volume and end systolic volume and an increase in the ejection fraction greater than 5%. The fact that the right and left SV did not increase significantly during exercise was consistent with the work of Belever et al. on adaptations of the heart during exercise in the supine position [42]. However, Bergovec et al. showed a significant increase in end diastolic volume and SV of the LV during exercise by radionuclide ventriculography [43]. More recent work by Stöhr et al. [44] confirmed that the SV increases early, at the beginning of the exercise, before reaching a plateau, after which the increase of CO is only related to the higher heart rate.

### Why exercise stress CMR could be interesting for RTOF

Early childhood surgery is essential for patient survival [4] but pulmonary regurgitation and stenosis are the major complication during follow up. Pulmonary regurgitation is often associated with other myocardial damage factors such as death, sustained ventricular tachycardia and increase in NYHA class. This is the leading cause of adverse events [2, 45, 46]. Revalvulation is the method of choice for reducing the symptoms and morbidity associated with RV damage secondary to pulmonary regurgitation, and it has been shown to be effective on the ventricular parameters [47]. This procedure can be performed either by endovascular or conventional surgery [48]. Some asymptomatic RTOF profiles have been identified as good responders. These patients would indeed not require further treatment as they are considered as definitively cured: Patients who are over 35 years old never having needed pulmonary valvulation with a normal exercise capacity and a pulmonary valve diameter greater than 0.5 multiplied by z score [49]. However, currently, we do not know the optimal timing for revalvulation. There is a consensus on the indication for valve replacement in symptomatic patients, but at this point it may already be too late and ventricular dilatation could be fixed [50–54]. To date, pulmonary revalvulation is based on morphological parameters such as RVEDV but these criteria are still a matter of debate [55–58].

### Aortic rigidity

In our study we showed that exercise unmasks a non-adaptation between AD and cardiac work in RTOF. That could be explained by the following hypothesis: surgery in childhood, aortic dextroposition or chronic ventricular damage may lead to secondary aortic wall damage. AD has been shown to be an early marker of damage to the smooth muscle fibers [59]. Measured lower adaptation to exercise might be an early sign of aortic rigidity increase and might corroborate with the higher incidence of aortic aneurysm and aortic wall histologic abnormalities in this population [60].

Therefore, the RTOF subjects probably fit into the framework of a more general cardiac condition as shown by the high number of cardiovascular events unrelated to RV damage [2, 45, 46]. Aortic rigidity is an important marker of risk for cardiovascular events, and increases faster with high mean blood pressure and a history of tobacco use [61, 62]. Aortic rigidity can cause changes in the geometry of the aortic arch and LV remodeling [63, 64] observed in the RTOF [65]. In comparison with the literature, Rutz et al. [66] found a lower AD at rest on AAo for RTOF compared to volunteers, suggesting an increased aortic rigidity in RTOF.

Our main limitation was a small number of subjects, a total of 22. However, Fallot's tetralogy remains a rare congenital heart disease, with an incidence of 1/3000 birth [14].

Another limitation was the fact that the image acquisitions were not carried out during exercise but less than a minute after pedaling stopped and with a short breath hold. We could not do better because the diameter of the magnet was too small to permit pedaling in the magnet. To compensate for this, exercise stress was monitored by the RPP calculation which showed a significant increase between rest and exercise and an average increase that was almost doubled in both the controls and the RTOF group, despite a broad dispersion. Selecting a control group using a paired matching procedure based on age and sex might have been helpful. VO2 consumption was not assessed and to the best of our knowledge there is no CE labeled device to do so in a CMR environment.

In conclusion, we have demonstrated the feasibility of performing an exercise test in an RTOF population under CMR condition within a reasonable time frame. With a dedicated protocol and a trained team this examination could be used in practice. In RTOF both ventricles were damaged by a lower contractile reserve. The ascending aorta showed early signs of increased rigidity during exercise, but not at rest.

Exercise CMR might be a promising tool for ventricular and aortic monitoring of RTOF.

## Supporting information

S1 File Patients data extracted from manipulations that allowed us to perform statistical analysis, comparison and reach our conclusions. (XLSX)

S2 File Volunteers data extracted from manipulations that allowed us to perform statistical analysis, comparison and reach our conclusions. (XLSX)

## Abbreviations

AC: aortic compliance
AD: aortic distensibility
AAo: ascending aorta
CMR: cardiac magnetic resonance
CO: cardiac output
DAo: descending aorta
LV: left ventricle
LVEF: left ventricular ejection fraction
RPP: rate pressure pulse
RTOF: repaired tetralogy of Fallot
RV: right ventricle
RVEF: right ventricular ejection fraction
SV: stroke volume
